# Single cell sequencing revealed the mechanism of PD-1 resistance affected by the expression profile of peripheral blood immune cells in ESCC

**DOI:** 10.3389/fimmu.2022.1004345

**Published:** 2022-11-17

**Authors:** Ting Deng, Huiya Wang, Changliang Yang, Mengsi Zuo, Zhi Ji, Ming Bai, Tao Ning, Rui Liu, Junyi Wang, Shaohua Ge, Le Zhang, Yi Ba, Haiyang Zhang

**Affiliations:** Key Laboratory of Cancer Prevention and Therapy, Tianjin, Tianjin’s Clinical Research Center for Cancer, National Clinical Research Center for Cancer, Tianjin Medical University Cancer Institute and Hospital, Tianjin, China

**Keywords:** single cell sequencing, PD-1 mMAB, peripheral blood, immune cells, ESCC

## Abstract

**Background:**

Esophageal squamous carcinoma (ESCC) is a highly lethal malignancy with poor prognosis. The effect of transcriptome characteristics of patient immune microenvironment (TME) on the efficacy of immunosuppressive agents is still poorly understood.

**Methods:**

Here we extracted and isolated immune cells from peripheral blood of patients with PD-1 monoclonal antibody sensitivity and resistance, and conducted deep single-cell RNA sequencing to describe the baseline landscape of the composition, lineage, and functional status of infiltrating immune cells in peripheral blood of patients with esophageal cancer.

**Results:**

The transcriptome characteristics of immune cells were comprehensively analyzed, and the dynamic changes of cell percentage, heterogeneity of cell subtypes and interactions between cells were explained. Co-expression and pedigree tracking based on T-cell antigen receptors revealed a significant proportion of highly migratory intertissue-effector T cells. GO and KEGG enrichment pathway Analysis of CD8^+^ effect-T cells ESCC_S group and ESCC_D1,2 group, found that in the up-regulated enrichment pathway, ESCC_S group enriched more PD-L1 and PD-1 checkpoint pathways expressed in tumors (JUN/NFKBIA/FOS/KRAS/IFNG), which also exist in T cell receptor signaling pathways. MT2A, MT1X and MT1E were differentially expressed in ESCC patients with PD-1 monoclonal antibody resistance, which may be related to the resistance of PD-1 mMAB.

**Conclusions:**

This study has an in-depth understanding of the influence of peripheral immune cell infiltration on the sensitivity of monoclonal antibody PD-1 in patients with esophageal cancer, which is helpful to promote the immunotherapy of patients with esophageal cancer.

## Introduction

Esophageal squamous carcinoma (ESCC) is one of the most challenging gastrointestinal tumors with high mortality (1). It was estimated to be the ninth most common cancer and the fifth leading cause of cancer death globally (2, 3). Current treatment options for patients with esophageal cancer include multidisciplinary management of local regional and locally advanced disease, and chemotherapy for palliative treatment of metastatic disease; however, esophageal cancer has a poor prognosis, with a low 5-year survival rate (4). With the widespread use of immune checkpoint inhibitors (ICIs), immunotherapy has made significant advances in the treatment of cancer patients. ICIs has several approved indications for the treatment of metastatic gastrointestinal malignancies, including gastric, esophageal, colorectal and hepatocellular carcinomas (5, 6).

PD-1 (programmed cell death -1) is a member of the CD28 cell surface receptor family that is expressed on activated T cells, B cells, NKT cells, monocytes, and macrophages, is crucial in regulating T -cell activation and tolerance (6, 7). The programmed death-ligand 1 (PD-L1)/PD-1 pathway is one of the most studied mechanisms of tumor immune escape (8). The successful development of PD-1 and PD-L1 monoclonal antibodies (mMAB) has led to significant advances in immunotherapy. Despite the clinical promise of ICIs, only a small proportion of patients with CI-responsive cancer subtypes benefit from treatment with anti-PD-1, PD-L1, and CTLA4 antibodies because of the high heterogeneity and complexity of cancer and the multiple mechanisms it uses to evade immune surveillance in addition to inhibiting anti-tumor T cell responses (9). It is of great significance to explore the mechanism of influencing the sensitivity of patients with esophageal cancer to PD-1 mMAB and to find effective biomarkers for predicting efficacy.

Various types of immune cells are present in peripheral blood, and studies have reported that they can predict the treatment response and clinical efficacy of anti-immune checkpoint inhibitors in patients with advanced cancer (10). The PBMC (Peripheral blood mononuclear cell) cell model, which includes T and B cells (~80%), natural killer cells (~10%), and monocytes (~10%), plays an important role in immune responses (11). Due to the availability and reproducibility of peripheral blood, it is more simple and feasible to study biomarkers of anti-tumor immunity by detecting and analyzing blood components compared with tissue samples (12). Therefore, the analysis of immune cells in peripheral blood of esophageal cancer patients is of great significance to explore the mechanism of PD-1 mMAB resistance in esophageal cancer.

Based on single cell data analysis, the expression of MT2A, MT1X and MT1E decreased in ESCC patients resistant to PD-1 monoclonal antibody, which may be related to PD-1 mMAB resistance. Metallothioneins (MTs) are small proteins rich in cysteine, which play an important role in metal homeostasis and prevention of heavy metal toxicity, DNA damage and oxidative stress. In humans, there are four main subtypes of Metallothioneins (MTs) (MT1, MT2, MT3, and MT4), which are encoded by a gene located on chromosome 16q13 (13). New evidence suggests that MTs play a key role in tumor formation, progression and drug resistance. However, MTs expression is not universal in all human tumors and may depend on tumor type and state of differentiation, as well as other environmental stimuli or genetic mutations.

MT2A stability triggers the apoptosis switch in stress response. XAF1 interacts directly with MT2A and promotes its lysosomal degradation, leading to elevated levels of free intercellular zinc, followed by p53 activation and XIAP inactivation. XAF1 is activated as a unique transcription target of metal-regulated transcription factor-1 (MTF-1) in signaling apoptosis, and its protein is unstable in the lysosomal pathway of MT2A induced by MTF-1 under cellular quiescent stress, indicating mutual antagonism between XAF1 and MT2A. Clinically, XAF1 and MT2A expression levels are negatively correlated in primary colon cancer and multiple cancer cell lines (14). Direct interaction of MT2A with BARD1 and BRCA1, co-localization of MT2A and BARD1 was detected by immunofluorescence. MT2A knockdown enhances oxaliplatin sensitivity in HT29 OR cells MT2A interacts with BARD1/BRCA1 and positively regulates and promotes oxaliplatin resistance in colorectal cancer cells (15). MT1X is considered as a tumor inhibitor, and is involved in the progression and metastasis of HCC (16). Expression and survival analysis showed that MT1X mRNA expression level was higher in normal tissues, which was associated with better prognosis of HCC patients (17). MT1E inhibits cell growth *in vitro* and *in vivo*, and MT1E can induce apoptosis of HCC cells and inhibit their metastasis. MT1E epigenetic silencing caused by promoter methylation may play an important role in HCC (18).

In this study, we performed single-cell RNA sequencing (scRNA-seq) to analyze peripheral blood immune cells from 4 patients with esophageal cancer who were treated with PD-1 mMAB. By comparing the peripheral blood immune cells of PD-1 mMAB sensitive and resistant patients, we comprehensively analyzed the transcriptomic characteristics of immune cells, and deciphered the dynamic changes of cell percentage, the heterogeneity of cell subtypes, and the interactions between cells, providing new knowledge for the biological basis of immunotherapy for esophageal cancer.

## Methods

### Human tissues dissociation and preparation

The tumor tissue samples of ESCC patients were obtained from Tianjin Medical University Cancer Institute and Hospital. As determined by clinical specialists, patients with PR (partial response, partial response was achieved with a reduction of ≥30% in the sum of maximum diameters of target lesions for at least 4 weeks) or SD (stable disease, the disease was stable, and the sum of maximum diameters of target lesions was not reduced to PR or enlarged to PD) after immunotherapy for more than half a year were classified as sensitive group, and patients with PD (progressive disease, disease progression, an increase of at least 20% in the sum of the maximum diameters of target lesions, or the appearance of new lesions) after immunotherapy for less than half a year were classified as drug-resistant group. We collected patients with advanced esophageal squamous cell carcinoma who were unresectable, sensitive or resistant to PD-1 mMAB, including two patients in each group. Their peripheral blood was collected and PBMCs were isolated for single-cell transcriptome sequencing. All patients provided informed consent, and Tianjin Medical University Cancer Institute and the hospital ethics Committee approved all aspects of the study (Ethics Approval Number: E2020169).

### Single cell RNA sequencing

The single-cell suspension with the concentration of 1×10^5^ cells/mL was prepared in PBS (HyClone). Single-cell suspensions were then loaded onto microfluidic devices and scRNA-seq libraries were constructed according to Singleron GEXSCOPER protocol by GEXSCOPER Single-Cell RNA Library Kit (Singleron Biotechnologies) (19). Individual libraries were diluted to 4nM and pooled for sequencing. Pools were sequenced on Illumina HiSeq X with 150 bp paired end reads.

### scRNA-seq quantifications and statistical analysis

The original reads are processed using an internal pipeline to generate gene expression profiles. Briefly, after filtering read one without poly T tails, cell barcode and UMI (unique molecular identifiers) was extracted. Adapters and poly A tails were trimmed (fastp V1) before aligning read two to GRCh38 with ensemble version 92 gene annotation (fastp 2.5.3a and featureCounts 1.6.2) (20). Reads with the same cell barcode, UMI and genes are grouped together to calculate the number of UMI per gene per cell. Using the same cell barcode, combine UMI and genes, and calculate the number of UMIs for each gene in each cell. Use the UMI count table of each cell’s barcode for further analysis.

Further analysis was performed using the UMI counting table for each cell bar code.

Reads with the same cell barcode, UMI and gene were grouped together to calculate the number of UMIs per gene per cell. The UMI count tables of each cellular barcode were used for further analysis. Cell type identification and clustering analysis using Seurat program (21, 22). The Seurat program (http://satijalab.org/seurat/ , R package,v.3.0.1 ) was applied for analysis of RNA-Sequencing data. UMI count tables were loaded into R using read.table function. Then we set the parameter resolution to 0.6 for FindClusters function to clustering analyses. Differentially expressed genes (DEGs) between different samples or consecutive clusters were identified with function FindMarkers. GO function enrichment analysis was performed on the gene set using the clusterProfiler software to find biological functions or pathways that are significantly associated with the genes specifically expressed (23).

### Primary analysis of raw read data

Raw reads from scRNA-seq were processed to generate gene expression matrixes using an internal pipeline. Briefly, raw reads were first processed with fastQC (24) v0.11.4 (https://www.bioinformatics.babraham.ac.uk/projects/fastqc/ ) and fastp (25) to remove low quality reads, and with cutadapt (26) to trim poly-A tail and adapter sequences. Cell barcode and UMI were extracted. After that, we used STAR (27) v2.5.3a to map reasds to the reference genome GRCh38 (ensembl version 92 annotation). UMI counts and gene counts of each cell were acquired with featureCounts (20) v1.6.2 software, and used to generate expression matrix files for subsequent analysis.

### Quality control, dimension-reduction and clustering

Before analyses, cells were filtered by UMI counts below 30,000 and gene counts between 200 to 5,000, followed by removing the cells with over 20% mitochondrial content. After filtering, the functions from Seurat v2.3 (22) was used for dimension-reduction and clustering. Then we used NormalizeData() and ScaleData funcitons to normalize and scale all gene expression, and selected the top 2000 variable genes with FindVariableFeautres function for PCA analysis. Using the top 20 principle components, we separated cells into multiple clusters with FindClusters. Batch effect between samples was removed by Harnomy (28). Finally, UMAP algorithm was applied to visualize cells in a two-dimensional space.

### Differentially expressed genes (DEGs) analysis

To identify differentially expressed genes (DEGs), we used the Seurat FindMarkers function based on Wilcox likelihood-ratio test with default parameters, and selected the genes expressed in more than 10% of the cells in a cluster and with an average log(Fold Change) value greater than 0.25 as DEGs. For the cell type annotation of each cluster, we combined the expression of canonical markers found in the DEGs with knowledge from literatures, and displayed the expression of markers of each cell type with heatmaps/dot plots/violin plots that were generated with Seurat DoHeatmap/DotPlot/Vlnplot function. Doublet cells were identified as expressing markers for different cell types, and removed manually.

### Multi-label immunofluorescence assay

(1) Put the tissue chips into the oven, set the temperature to 63 degrees, and bake for one hour.
(2) Dewaxing is completed in the automatic dyeing machine, and the dewaxing time is as follows:

Two cylinders of xylene, each 15 minutes; 2 jars of 100% alcohol, 7 minutes each; 90% alcohol 1 jar, 5 minutes; One jar of 80% alcohol, 5 minutes; One jar of 70% alcohol, 5 minutes. (3) Antigen repair: Dilute 10 repair solution to 1× working solution, microwave oven to high heat for 3min and boil, then put in the glass slides, microwave oven power to low heat to continue repair for 15-20min (ensure that the tissue is immersed in liquid during the whole process), cool naturally at room temperature, and soak the glass slides in pure water. (4) Remove endogenous peroxidase:

The slides were removed, placed in a wet box, treated with commercial H2O2 for 10min, and cleaned with TBST. (5) The slides were taken out and placed in a wet box, then blocking buffer was dropped and incubated for 10min. (6) Primary antibody incubation: Blocking buffer was removed, diluted primary antibody working solution was dropped, incubated at room temperature for 1h, and the slides were cleaned by TBST. (7) The slides were removed, placed in a wet box, dropped secondary antibody, incubated at room temperature for 10min, and cleaned by TBST. (8) The slides were removed and placed in a wet box. Opal dye diluent (dilution ratio: 1:100) was dropped and incubated at room temperature for 10min. The slides were cleaned by TBST. (9) Dilute 10× repair solution to 1× working solution, microwave oven to high heat for 3min and boil, then put in glass slides, microwave oven power to low heat to continue repair for 15-20min (ensure that the tissue is immersed in liquid during the whole process), cool naturally at room temperature, TBST clean the glass slides. (10) DAPI dyeing and sealing.

### Pathway enrichment analysis

To investigate the potential functions of DEGs, the Gene Ontology (GO) and Kyoto Encyclopedia of Genes and Genomes (KEGG) analysis were used with the “clusterProfiler” R package (23). Pathways with p_adj value less than 0.05 were considered as significantly enriched. Gene Ontology gene sets including molecular function (MF), biological process (BP), and cellular component (CC) categories were used as reference. Protein-protein interactions (PPI) of DEGs in each cluster were predicted based on known interactions of genes with relevant GO terms in the StringDB v1.22.0.

### Gene regulatory network inference

To analyze transcription factor regulatory networks, we performed SCENIC R toolkit (29) using scRNA expression matrix and transcription factors in AnimalTFDB. Regulatory networks were predicted by the GENIE3 package based on the co-expression of regulators and targets. We used the RcisTarget package to search for transcription factor binding motifs in the data. Genes involved in a predicted regulatory network were defined as a gene set, whose auc value was calculated by the AUCell package to assess the activity of the regulatory network in cells.

### Trajectory analysis

Cell differentiation trajectory was reconstructed with the Monocle2 (30). Differentially expressed genes were used to sort cells in order of spatial‐temporal differentiation. We used DDRTree to perform FindVairableFeatures and dimension-reduction. Finally, The trajectory was visualized by plot_cell_trajectory() function.

## Results

### Single-cell transcriptome analysis of peripheral immune microenvironment in ESCC

**Figure 1:**
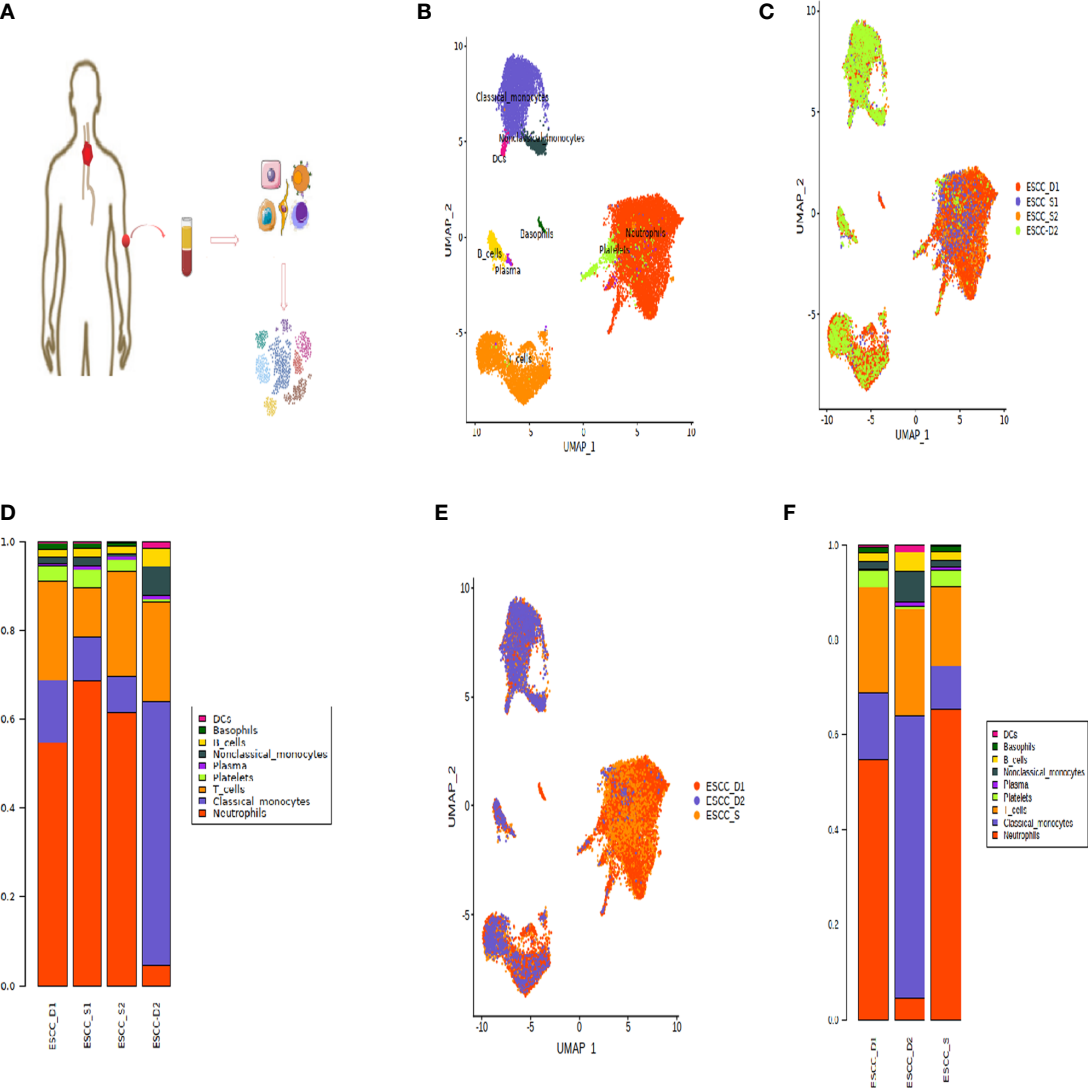
Peripheral blood single cell atlas of patients with esophageal cancer. **(A)** Workflow showing the scRNA-seq experimental design and initial data exploration. **(B, C)** UMAP plot of 18,121 high-quality immune cells to show nine main cell-types based on the expression of known marker genes, colored by cell type and cell origin respectively. **(D)** The proportion of cells that contributed to each cluster by each sample, colored by cell types. **(E)** UMAP plot of 18,121 high-quality immune cells to show nine main cell-types based on the cell sources. **(F)** The proportion of cells that contributed to each cluster.

We collected 4 patients with advanced esophageal squamous cell carcinoma who were surgically unresectable and treated with PD-1 mMAB. According to their sensitivity to PD-1 mMAB, they were divided into sensitive and drug-resistant groups, abbreviated as ESCC-S and ESCC-D, respectively, and then replaced them with the abbreviation (**Table 1**). Their peripheral blood was collected and PBMCs were isolated for single-cell transcriptome sequencing to explore the cellular characteristics of TME (**Figure 1A**). After initial quality control assessment and dual body removal, we obtained single-cell transcriptomes totaling 19878 cells, total number of genes identified ranged from 24274 to 30512 per cell, with an average of 27099 for the detected genes. The single-cell map of immune cell transcriptome in peripheral blood of ESCC was characterized, and the differential characteristics of subsets and gene expression in sensitive and drug-resistant groups were analyzed. By graph-based uniform manifold approximation and projection (UMAP), 9 high-confidence cell clusters were identified to show main cell-types based on the expression of known marker genes. In particular, they were as follows: neutrophils, classical monocytes, T cells, platelets, plasma, nonclassical monocytes, B cells, basophils, dendritic cells (DCs). Cell clustering is manually annotated based on marker genes of each cell type, as shown in the **Table 2**.

**Table 1:** Basic information of patients with ECSS.

Number	Sample Name	Species	Age	Gender	TNM Stages	Therapeutic Regimen	Sensibility
1	ESCC_S1	human	65	Male	IIIB	Paclitaxel + cis-platinum + PD-1 mMAB	Yes
2	ESCC_S2	human	55	Male	IVB	Paclitaxel + cis-platinum + PD-1 mMAB	Yes
3	ESCC_D1	human	54	Male	IVA	Paclitaxel + cis-platinum + PD-1 mMAB	No
4	ESCC-D2	human	60	Male	IVA	Paclitaxel + cis-platinum + PD-1 mMAB	No

**Table 2:** Cell types and corresponding markers.

Cell type	Abbreviation	Marker
T cells	T cells	CD3D,NKG7,IL7R
B cells	B cells	MS4A1,CD79A
Classical monocytes	Classical_monocytes	CD14,VCAN,FCN1
Nonclassical monocytes	Nonclassical_monocytes	FCGR3A,FCN1,IFITM3
Dendritic cells	DCs	CD1C,FCER1A
Neutrophils	Neutrophils	CSF3R,CXCR2,FCGR3B
Basophils	Basophils	CLC,CPA3
Platelets	Platelets	PPBP,PF4

The dimension reduction UMAP by cell type and cell source is shown in the **Figures 1B, C**. The bar chart shows the proportion of each cell type (**Figure 1D**). According to the bar chart, it can be seen that the proportion of each cell type in the samples of the two sensitive groups is similar. Therefore, for the convenience of subsequent data analysis and comparison, we combined the sensitive group data into one group (**Figure 1E**). However, the proportion of all types of cells in D1 and D2 was quite different, so they were not combined in order to find out the difference.

**Figure 2:**
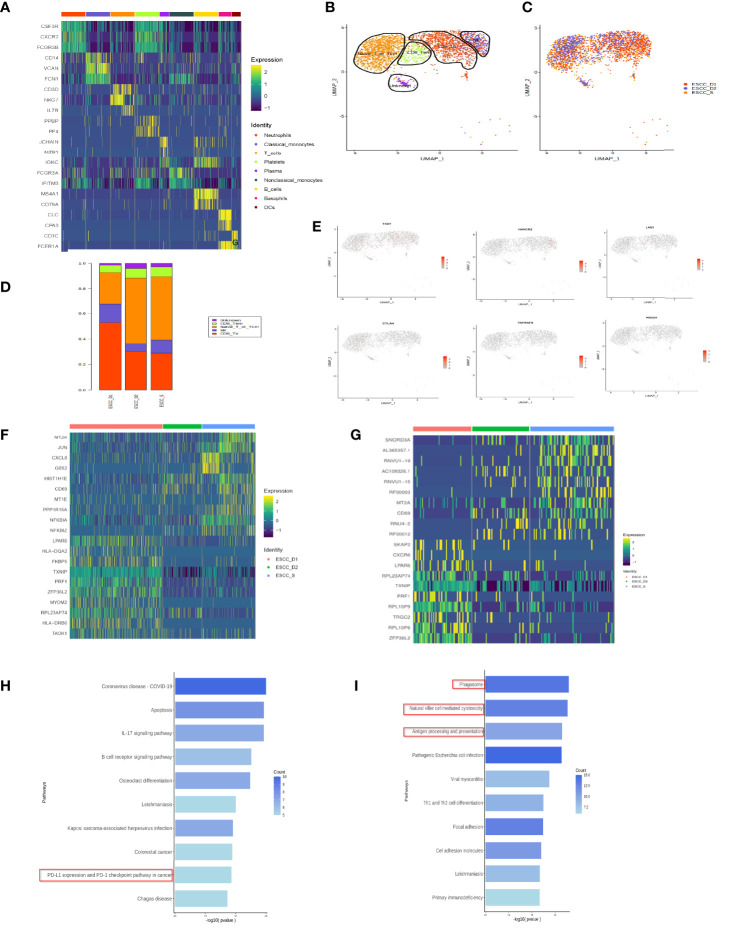
T cell subsets in peripheral blood of esophageal carcinoma. **(A)** Heatmap showing the relative expression level of genes across cells which were used to identify cell types. **(B)** UMAP showing the clustering of T cell subsets based on the expression of marker genes. **(C)** UMAP showing the merge T cells from ESCC patients. Each dot corresponds to one single cell colored according to cell cluster. **(D)** The proportion of cells that contributed to each cluster. **(E)** The expression of signature genes in exhausted T cells. T cells are less or do not express the marker genes of exhausted T cells. **(F)** Heatmap showing the relative expression level of genes between PD-1 mMAB resistant and sensitive ESCC patients in CD8^+^ T effective cells. **(G)** Heatmap showing the relative expression level of genes between PD-1 mMAB resistant and sensitive ESCC patients in CD8^+^ T memory cells. **(H)** GO and KEGG enrichment analysis of ESCC_S group and ESCC_D1, 2, CD8^+^ effector T cell group up-regulated differential gene enrichment pathways. **(I)** GO and KEGG enrichment analysis of ESCC_S group and ESCC_D1, 2, CD8^+^ effector T cell group down-regulated differential gene enrichment pathways.

According to the proportion of cells after cell clustering, three distinct cell subpopulations were selected, including T cells, monocytes and neutrophils (**Figure 1F**). Heatmap showing the relative expression level of genes across cells which were used to identify cell types (**Figure 2A**).

### A single-cell atlas of T cells in peripheral blood of esophageal carcinoma

According to previous literature reports and cell marker gene annotation database, T cells were subdivided into four cell types, including effector T (Te), effector memory T (Tem), initial or central memory T and nature kill (NK) cells (**Figures 2B–D**, **Table 3**). Through the analysis of differential genes, no corresponding cell subgroup was found, so we defined it as the unkown group (**Figures 2B–D**). In addition, we wanted to know whether there were exhausted T cells in the peripheral blood. As shown in the **Figure 2E**, we compared the marker of exhausted T cell, and found that marker genes TIGIT and TIM3 were less expressed, and the other 4 markers were basically unexpressed (**Figure 2E**).

**Table 3:** T cell subsets and corresponding markers.

Cell type	Abbreviation	Marker
CD8+effector T cells	CD8_Te	CD3D,CD8A/B,NKG7,GNLY
CD8+ effector memory T cells	CD8_Tem	CD3D,CD8A/B,GZMK,KLRG1,IL7R
Naïve T cells/ Central memory T cells	Naive_T_or_Tcm	CD3D,CCR7,TCF7,IL7R
NK cells	NK	CD3D-,TRDC,KLRD1,KLRF1

### Pseudo time sequence analysis of T cell subsets

Based on the changes in gene expression levels of peripheral blood T cell subsets over time, the cell lineage development was constructed (**Supplementary Figures 1A**). There are two developmental branches on the pseudo-temporal sequence of quasi-temporal analysis, from naive T cells and memory T cells to effector T cells (**Supplementary Figures 1B**). The distribution of each cell type alone on a quasi - time trajectory is also shown in **Supplementary Figures 1C**. The distribution diagram of each sample in the pseudo timing trajectory, in which different colors represent the cell types in each sample, and the results of pseudo timing analysis of confluent cells (**Supplementary Figures 1D**). With the dynamic change of pseudo time, gene expression changes with pseudo time (**Supplementary Figures 1E**). The expression of the first 8 genes in reverse order of Q value varies with the change of pseudo time (**Supplementary Figures 1F**)

The results of pseudo time sequence analysis of T cell subsets showed the differentiation of three T cell subsets (**Supplementary Figures 1A**), the differentiation and development trajectories of T cell subsets simulated according to time change, and the development trajectories of T cells from different sample sources (**Supplementary Figures 1C**). We observed that most of Naive_T_or_Tcm cells differentiated into CD8^+^ effector T cells (CD8_Te cells) and only a small part differentiated into (CD8_Tem cells) in peripheral blood of ESCC. The ESCC_D1 group mainly differentiated into CD8^+^ effector T cells in the middle and late stage of differentiation, and the ESCC_D2 group mainly differentiated into CD8^+^ effector T cells in the early stage of differentiation. In the ESCC_S group, Naive_T_or_Tcm cells differentiated into CD8^+^ effector T cells in the whole process.

The dynamic change trend of gene expression (**Supplementary Figures 1F**) shows three gene expression patterns in the process of T cell differentiation. Cluster1 represents the gene group with decreasing expression along with the process of T cell differentiation, Cluster2 represents the gene group with increasing expression. Cluster3 indicates the presence of both up-regulated and down-regulated gene groups. In addition, we show the dynamic changes in the expression of some genes during differentiation. As shown in **Supplementary Figures 1G**, chemokine CCL5 was gradually up-regulated when Naive_T_or_Tcm cells began to differentiate, and then its expression gradually became stable after differentiation into CD8^+^ effector T cells. Genes related to cytotoxicity, such as NKG7, GNLY and CST7, began to be up-regulated in CD8^+^ effector T cells at the early stage of differentiation, and gradually increased with the extension of differentiation time. Other genes related to cytotoxic status, such as GZMB and PRF1, were up-regulated at late differentiation stage.

### Differences of peripheral blood T cells between patients with sensitivity and drug resistance to PD-1 mMAB in esophageal cancer

The proportion of CD8^+^ effector T cells in ESCC_D1 group was the highest, compared to ESCC_S group. The proportion of CD8^+^ memory T cells was the lowest, and there was no significant difference between ESCC_D1,2 group and ESCC_S group. ESCC_S group had the highest proportion of primary/juvenile T cells, which was higher than ESCC_D1,2 group (**Figure 2D**). Heatmap showing the relative expression level of genes between PD-1 mMAB resistant and sensitive ESCC patients in CD8^+^ T effective cells and CD8^+^ T memory cells (**Figures 2F, G**). GO and KEGG enrichment pathway Analysis of CD8^+^ effect-T cells ESCC_S group and ESCC_D1,2 group, found that in the up-regulated enrichment pathway, ESCC_S group enriched more PD-L1 and PD-1 checkpoint pathways expressed in tumors (JUN/NFKBIA/FOS/KRAS/IFNG), which also exist in T cell receptor signaling pathways. In the down-regulated enrichment pathway, the genes related to phagocytic NK cell-mediated cytotoxicity and antigen presentation were enriched in group ESCC_D1,2. CD8^+^ memory T cells ESCC_S group and ESCC_D1 group were also enriched in the down-regulated enrichment pathway of NK cell-mediated cytotoxicity and antigen presentation and other related genes. KEGG pathway enrichment analysis showed that compared with ESCC_D1 group, ESCC_S group was enriched in more PD-L1 and PD-1 checkpoint pathways expressed in tumors (JUN/NFKBIA/FOS/KRAS/IFNG), as well as B-cell receptor signaling pathways (**Figure 2H**).

In addition, genes with enriched up-regulated pathways in the ESCC_D1 group were mainly related to phagocytic and NK cell-mediated cytotoxicity and antigen presentation (**Figure 2I**).

### MT2A, MT1E and MT1X were differentially expressed in PD-1 mMAB resistant ESCC patients

**Figure 3:**
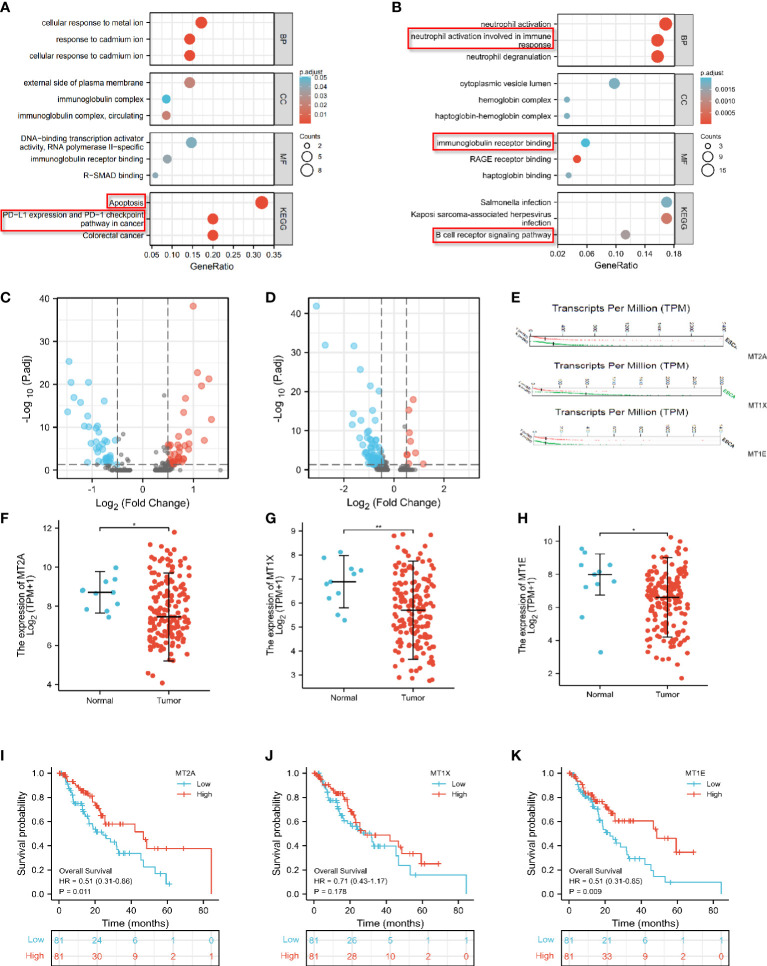
Differential expression analysis of MT2A, MT1X and MT1E. **(A)** GO and KEGG enrichment analysis of genes with significantly lower expression in PD-1 mMAB resistant patients ESCC-D1 compared with sensitive patients ESCC-S. **(B)** GO and KEGG enrichment analysis of genes with significantly lower expression in PD-1 mMAB resistant patients ESCC-D2 compared with sensitive patients ESCC-S. **(C)** The volcano map shows the differentially expressed genes in ESCC_D1 patients compared with ESCC_S patients. **(D)** The volcano map shows the differentially expressed genes in ESCC_D2 patients compared with ESCC_S patients. **(E)** GEPIA database analysis, MT2A, MT1E, MT1X molecule transcription levels in patients with esophageal cancer and normal controls. **(F–H)** TCGA database analysis, MT2A, MT1E, MT1X molecule expression levels in patients with esophageal cancer and normal people. **(I–K)** TCGA database was used to analyze the prognosis and survival of patients with high and low expression of MTA2, MT1E and MT1X in esophageal cancer patients. *p < 0.05, **p< 0.01, respectively.

KEGG enrichment analysis of ESCC_S group and ESCC_D1 group showed that decreased gene expression in D1 group was related to cell apoptosis and PD-L1 expression (**Figure 3A**). ESCC_D2 compared with ESCC_S group and neutrophils activated to participate in the immune response, and the expression of immunoantigen presentation related genes decreased. ESCC_D2 compared with ESCC_S-enriched B cell receptor signaling pathway related pathways decreased (**Figure 3B**). In order to explore the role of differential genes in promoting PD-1 mMAB resistance in ESCC, we compared the differential genes in ESCC_D1 and ESCC_D2 contrast sensitive patients (**Figures 3C, D**), and detected the differentially expressed genes in both resistant patients compared with sensitive patients by Venn map (**Supplementary Figures 1H, I**).

Among these differential genes, MT2A, MT1E and MT1X have attracted our attention. Compared with the sensitive group, the expression of MT2A, MT1E and MT1X in ESCC_D1 and ESCC_D2 patients was decreased, with statistical significance (p<0.001, p<0.05, p<0.05). These three molecules belong to the Metallothioneins family, and previous studies have shown that they play a tumor suppressor role in some malignant tumors, but there are few studies in esophageal cancer. GEPIA database analysis, MT1E, MT1X molecule transcription levels in esophageal cancer patients and normal control group MT2A, MT1E, MT1X molecule (**Figure 3E**). The expression of MT2A, MT1E and MT1X in esophageal cancer patients and normal persons was detected by TCGA database, and it was found that the expression of MT2A, MT1E and MT1X in esophageal cancer patients was significantly reduced (**Figures 3F–H**). TCGA database was used to analyze the relationship between expression of MTA2, MT1E and MT1X in esophageal cancer and prognosis and survival (**Figures 3I–K**, **Supplementary Figure 1J**), and the analysis results showed that low expression of MT2A, MT1E and MT1X was associated with poorer overall survival.

**Figure 4:**
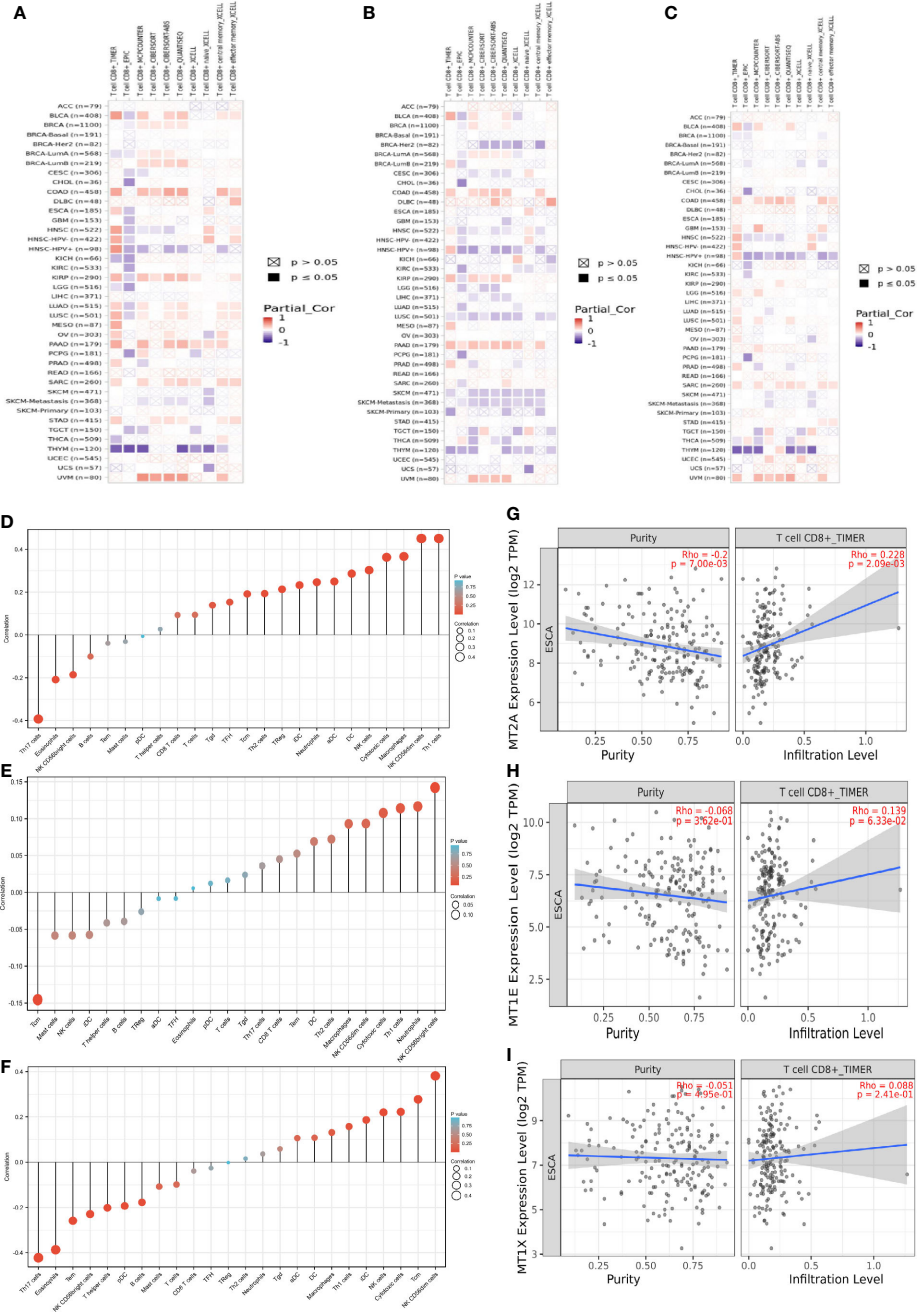
Prognostic correlation analysis of MT2A, MT1X and MT1E. **(A–C)** The correlation between MT2A, MT1E, MT1X expression and CD8^+^ T cells in various malignancies was analyzed by various databases. **(D–F)** TIMER database was used to analyze the relationship between MT2A, MT1E, MT1X expression and various immune cell infiltration in ESCC. **(G–I)** The relationship between MT2A expression and CD8^+^ T cell infiltration in ESCC was analyzed by TIMER database.

Next, the correlation between MT2A, MT1E, MT1X expression and CD8^+^ T cells and other immune cells in various malignant tumors was analyzed by bioinformatics database (**Figures 4A–C**).

MT2A, MT1E, MT1X expression is associated with CD8^+^ T cell infiltration in malignant tumors including esophageal cancer. TIMER database was used to analyze the relationship between the expression of MT2A, MT1E and MT1X in ESCC and the infiltration of T cells, B cells, natural killer cells, dendritic cells, macrophages and other immune cells (**Figures 4D–F**). The expression of MT2A, MT1E and MT1X in esophageal carcinoma and their relationship with CD8^+^ T cell infiltration were detected (**Figures 4G–I**).

**Figure 5:**
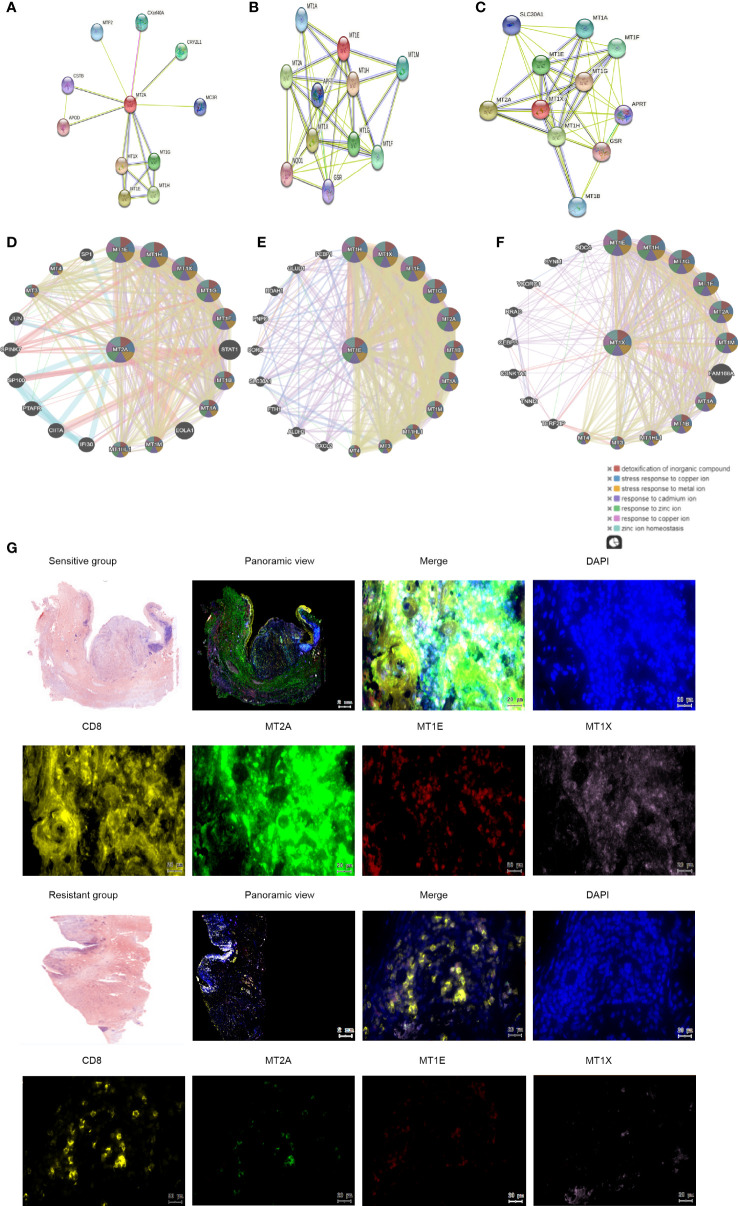
Molecular interactions of MT2A, MT1X and MT1E. **(A–C)** String database analysis of genes interacting with MT2A, MT1E and MT1X. **(D–F)** GeneMANIA database was used to analyze the interaction between MT2A, MT1E and MT1X. **(G)** The expression of MT2A, MT1E and MT1X in PD-1 mMAB sensitive and resistant esophageal cancer tissues was detected by multicolor immunohistochemical assay (n=3).

Sting database analysis of molecular interactions of MT2A, MT1E and MT1X showed that interactions with other molecules in the Metallothioneins family (**Figures 5A–C**). GeneMANIA database analysis of MT2A, MT1E, MT1X analysis of the interaction of molecules, and showed correlations with detoxification of inorganic compound,stress response to copper ion and metal ion (**Figures 5D–F**). In order to further identify the key factors related to drug resistance of PD-1 mMAB in esophageal cancer, we performed Multi-label immunofluorescence assay validation on tissue sections of PD-1 mMAB sensitive and PD-1 mMAB resistant samples of ESCC patients after anti-PD-1 treatment. The results of Multi-label immunofluorescence assay showed that compared with the tissues of sensitive patients, the proportion of CD8^+^ T cells positive for MT2A, MT1E and MT1X was less in the esophageal cancer tissues of resistant patients, which were consistent with the results of single cell sequencing (**Figure 5G**, **Supplementary Figures 1K, L**).

These results indicated that the expression of MT2A, MT1E and MT1X in CD8^+^ T cells of PD-1 mMAB resistant ESCC patients is decreased, which may be related to the poor sensitivity of PD-1 mMAB.

### Analysis of single cell subsets of monocytes in peripheral blood of esophageal carcinoma

**Figure 6:**
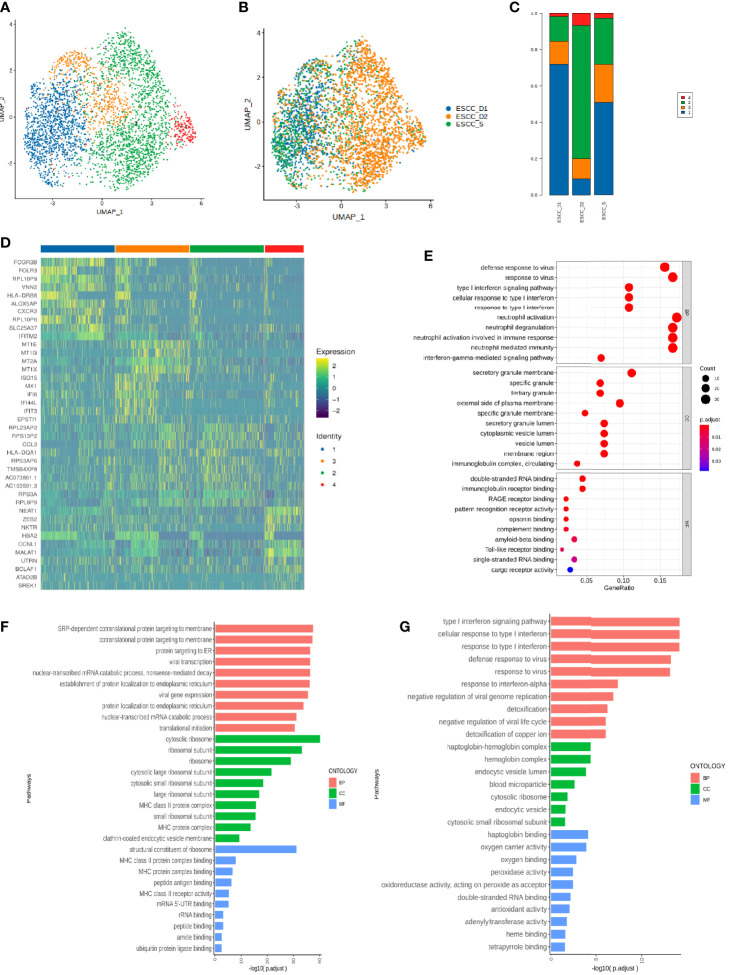
Single-cell subsets of mononuclear cells in peripheral blood of ESCC. **(A)**UMAP showing the clustering of monocyte subsets based on the expression of marker genes. Each dot corresponds to one single cell colored according to cell cluster. **(B)** UMAP showing the merge monocyte cells from ESCC patients. **(C)** The proportion of cells that contributed to each cluster by each sample. **(D)** The heat map shows the relative expression levels of differential genes in each subtype of monocytes. **(E–G)** GO enrichment analysis of monocyte Cluster 1-3.

Monocytes were subdivided into 4 cell clusters in single-cell analysis, which could not be defined according to existing regulations, so they were temporarily named as Cluster 1-4 (**Figure 6A**). The proportion of Cluster 1 and Cluster 2 was relatively high, and the proportion of Cluster 3 cells in the resistant group was lower than that in the sensitive group (**Figures 6B, C**). We also identified specific gene sets for these cell subpopulations to allow more in-depth analysis of regulatory pathways. The list of differential genes in each cluster of monocytes was selected and sorted in descending order of avg_logFC, and the top 10 genes were selected for heat map drawing, in which duplicate genes would be removed (**Figure 6D**). GO enrichment analysis and KEGG pathway enrichment analysis were performed for each cluster. The Cluster 1 enrichment analysis showed that the differential genes with decreased expression in the resistant group compared with the sensitive group were related to type I interferon signaling interferon, neutrophil activation, neutrophil degranulation (**Figure 6E**, **Supplementary Figures 2A**). Cluster 2 is related to protein targeting to the membrane, localization to the endoplasmic reticulum and mRNA decomposition (**Figure 6F**,**Supplementary Figures 2B**). Cluster 3 is mainly enriched in type I interferon signaling pathway and associated with virus infection. Significantly enriched genes included MX1 or 2/ISG15/OAS3/IFI6/IFIT2 or 3/XAF1/OAS1/IFI35/IFITM3 (**Figure 6G**, **Supplementary Figures 2C**). Cluster 4 is related to mRNA/RNA splicting (**Supplementary Figures 2D, E**).

Type I interferon is key driver of inflammation and immune suppression in chronic infections, providing essential inflammatory signals, however, initiate feedback suppression in immune cells and cancer cells (31). The enrichment pathway of monocytes subsets in PD-1 mMAB resistant patients is related to type I interferon, which may be related to the reduction of PD-1 mMAB sensitive.

### Heterogeneity analysis of single cell subsets of neutrophil in peripheral blood of esophageal carcinoma

**Figure 7:**
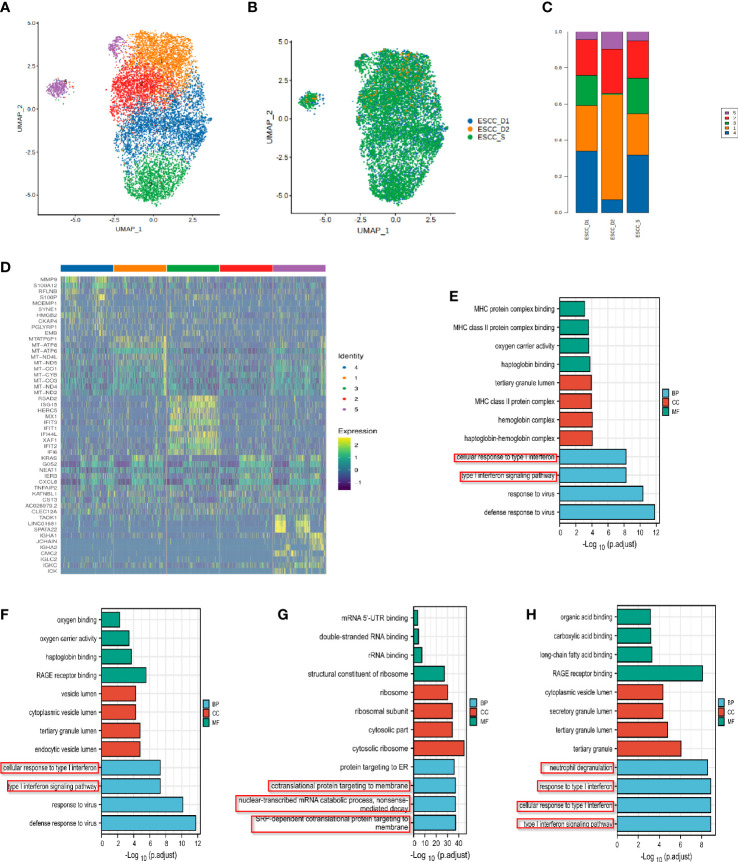
Analysis of single cell subsets of neutrophils in peripheral blood of ESCC. **(A)** UMAP showing the clustering of neutrophils subsets based on the expression of marker genes. **(B)** UMAP showing the merge neutrophils from ESCC patients. **(C)** The proportion of cells that contributed to each cluster by each sample. **(D)** The heat map shows the relative expression levels of differential genes in each neutrophil cluster. **(E)** Neutrophil cluster 1 GO enrichment analysis showed that it was related to MHC class II molecular antigen presentation process. **(F–H)** GO enrichment analysis of neutrophil Cluster 2-4.

Neutrophils were further subdivided into 5 clusters for further analysis (**Figure 7A**). Neutrophil Cluster 1-4 accounted for a higher proportion, while Cluster 5 accounted for a smaller proportion (**Figures 7B, C**). The list of differential genes in different subpopulations of monocytes was sorted in descending order of avg_logFC, and the top 10 genes were selected for heat mapping, in which duplicate genes would be removed (**Figure 7D**). The proportion of Cluster 1 neutrophils was the highest and the differential genes with reduced expression in the resistant group compared with the sensitive group were significantly correlated with the antigen presentation process of MHC class II molecules in the immune response, the cellular response of type I interferon and the type I interferon signaling pathway (**Figure 7E**, **Supplementary Figure 3A**). The enrichment function of Cluster 2 is mainly related to the cellular response of type I interferon and the type I interferon signaling pathway (**Figure 7F**, **Supplementary Figure 3B**). This suggests impaired anti-tumor function such as neutrophil antigen presentation recognition in patients with PD-1 mMAB resistance. GO enrichment analysis of Cluster 3 showed that the gene with reduced expression in the resistant group was associated with defense response to virus and type I interferon signaling pathway (**Figure 7G**, **Supplementary Figure 3C**). The results of differential gene enrichment analysis in Cluster 4 is related to chemotaxis of neutrophils and DCs, cytokine release and T cell-mediated cytotoxicity (**Figure 7H**, **Supplementary Figure 3D**). Cluster 5 is involved in protein targeting to membranes, endoplasmic reticulum, and ribosomal assembly (**Supplementary Figures 3D, E**).

The enrichment pathway of neutrophil subsets in the resistant group was also related to type I interferon, and was related to the chemotaxis of neutrophils and DCs, which may play a synergistic role with monocytes in the PD-1 mMAB resistant of esophageal cancer.

### Regulation analysis of single cell subsets of transcription factors

The regulation of transcription factors on gene expression of single cell subsets was further explored, AUC scores of transcription factors and target gene sets were shown in the heat map (**Supplementary Figures 4A**). Top10 Regulon’s AUC matrix clustering heat map in each cell is shown in **Supplementary Figures 4B**. Taking effector T cells as an example, regulon-specific scatter plots of sensitive and drug-resistant patients were shown, highlighting the highest top 10 Regulon (**Supplementary Figures 4C-E**). According to the Venn diagram results, ESCC_D2 had four transcription factors specifically expressed higher than the other two samples (**Supplementary Figures 4F**). Among them activated T nuclear factor (NFAT) has been identified for the first time as a major stimulus-responsive DNA binding factor and transcriptional regulator in T cells (32). NFAT transcription factors are assumed to play a central role in the carcinogenesis of pancreatic cancer (33). Array analysis showed that NFATc2 was the c-REL target gene among the 12 trail inducing genes that were the strongest in apoptotic resistant cells (34). NFAT may play an important role in resistance to PD-1 mMAB in esophageal cancer.

## Disscusion

ESCC is the leading malignant tumor worldwide, accounting for about 572,000 new patients and 508,000 deaths annually (35). ESCC usually contains extensive genomic changes, and although there are exceptions, a high mutation load is associated with a better response to checkpoint blockade. A number of large phase II/III clinical trials targeting the first and second lines of advanced esophageal cancer have confirmed that immunotherapy brings significant clinical benefits to patients with advanced esophageal cancer (36, 37).

PD-1 belongs to the CD28 cell surface receptor family and is expressed on activated T cells, B cells, NKT cells, monocytes and macrophages. Its ligand, PD-L1, is upregulated in many cancers and is an important target in immunotherapy of tumors (38, 39). Anti-programmed death 1 (PD-1)/programmed death ligand-1 (PD-L1) therapy shows antitumor activity in patients with metastatic esophageal cancer (40). In a randomized Phase III study of Keynote-181, embrolizumab extended overall survival (OS) in patients with advanced esophageal cancer compared with chemotherapy as second-line treatment, compared with programmed death ligand 1 (PD-L1) combined positive score (CPS)≥10 (41). Nivolumab was associated with a significant improvement in overall survival and favorable safety compared to chemotherapy in previously treated patients with advanced esophageal squamous cell carcinoma, and may represent a new standard second-line treatment option for these patients (42). While in Keynote-590, drug K combined with first-line chemotherapy for the whole population brought significant OS improvement regardless of PD-L1 expression (43). Other studies have also found that immunotherapy has a better effect on patients with PD-L1 CPS≥ 10, but some patients can still benefit from PD-L1 CPS < 10. Therefore, how to screen immunotherapy population is very important. In other words, PD-L1 is not the only population screening marker, and more markers need to be found to guide immunotherapy, so as to develop individualized and precise treatment plans.

Recently, scRNA-seq has been developed to untargeted quantification of the transcripts present in individual cells (44). Advances in molecular biology, microfluidics and bioinformatics have empowered the study of thousands or even millions of individual cells from malignant tumors at the single-cell level of resolution (45). The use of single-cell sequencing in cancer research has revolutionized our understanding of the biological features and dynamics within cancer lesions (46). The ability to find and characterize abnormal cells in the population has potential implications for further understanding of drug resistance and recurrence in cancer therapy (47). At present, an increasing number of tumor studies, including a variety of solid tumors, such as malignant melanoma (48), lung cancer (49, 50), breast cancer (51), straight colon cancer (52), gastric cancer (53), esophageal cancer (37) and pancreatic cancer (54), have used single-cell sequencing technology to map tumor immune cells. The interaction between tumor and immune system can be comprehensively evaluated by visual method, which can be used to predict the efficacy of immunotherapy.

We sequenced peripheral blood of 4 patients with esophageal cancer by single-cell sequencing technology to explore the influence of immune cell gene differences on cancer PD-1 sensitivity of esophageal patients. Compared with ESCC_S group, ESCC_D1 group had the highest proportion of CD8^+^ effector T cells. Analysis of CD8^+^ effect-T cells ESCC_S group and ESCC_D1 group showed that among the up-regulated enrichment pathways, ESCC_S group enriched more PD-L1 and PD-1 checkpoint pathways (JUN/NFKBIA/FOS/KRAS/IFNG) expressed in tumors. These pathways are also present in T cell receptor signaling pathways. MT2A, MT1E and MT1X were differentially expressed in ESCC patients resistant to PD-1 mMAB. The expression of MT2A, MT1E and MT1X in esophageal cancer patients and normal controls was detected by TCGA database, and it was found that the expression of MT2A, MT1E and MT1X in esophageal cancer patients was significantly reduced, which was associated with poor prognosis. Metallothionein is a cysteine rich cytoplasmic protein with low molecular weight (6-7 kDa), which plays an important role in metal ion homeostasis and detoxification (55). In recent years, many studies have shown that MTs expression is different in different tumors, suggesting that MTs may play an important role in tumorigenesis (13, 56). MTs expression is not universal in all human tumors, and may play different or even opposite roles in different tumors. A Japanese researcher found that MT2A was highly expressed in CAF cells constructed by them. Knockdown of MT2A inhibited the expression and secretion of insulin-like growth factor binding protein 2 (IGFBP2), and recombinant IGFBP2 promoted the migration and invasion of ESCC cells through NF-κB, Akt and Erk signaling pathways (57). The opposite effect of MTs in different tumors may be related to tumor type and differentiation, other environmental stimuli or gene mutations (13).

MT2A, MT1E, MT1X are identified as potential novel therapeutic targets in ESCC. Compared with the sensitive group, MT2A, MT1E, MT1X expression were down-regulated in immune-resistant patients, and were correlated with the infiltration of various immune cells, including CD8^+^ effector T cells, in tumor tissues. Therefore, MT2A, MT1E, MT1X may be potential predictors of anti-PD-1 therapy in patients with advanced esophageal squamous cell carcinoma.

Our results also found that differences in T cell subtype characteristics between immunotherapy response and nonresponse groups could not be determined solely by the proportion of cell subtypes, but circulating CD8^+^ effector T cells were the dominant cell subsets in both response and nonresponse groups. Similar findings have been reported in other solid tumors. For example, a study reported that after anti-PD-1 inhibitor treatment in melanoma patients, the frequency of CD8 effector memory T cells in the circulating blood of responders increased, while the frequency of CD4 effector memory T cells and CD8 naive T cells decreased (58). In recent years, more and more researchers have discovered the disturbance of ICIs on tumor microenvironment and circulating immune cells in peripheral blood by single cell sequencing technology. Overall, the difference in immune cells is mainly between T cells. Among T lymphocytes, cytotoxic CD8^+^ T cells are usually affected by ICIs treatment, and they play a huge role in tumor monitoring, editing and control (59).

Monocytes and neutrophils are also important immune cells in peripheral blood. Reduced PD-1 expression on peripheral blood T cells and reduced monocyte populations in the glioblastoma tumor microenvironment were more frequent in the neoadjuvant group than in patients treated only in the neoadjuvant group (60). Studies have shown that the endogenous microbiome in pancreatic cancer promotes tumorigenesis by differentially activating Toll-like receptors selected in monocytes to generate tolerance immune programs (61). Interleukin-17 in pancreatic cancer recruits neutrophils, triggers neutrophil extracellular traps (NETs), and excludes cytotoxic CD8 T cells from the tumor, reducing the sensitivity of immune checkpoint blockade (PD-1, CTLA4) (62). Elevated serum interleukin-8 and enhanced intratumoral neutrophil infiltration are associated with poorer prognosis in advanced cancer and reduce the clinical benefit of immune checkpoint inhibitors (63). We found that the enrichment pathway of monocyte and neutrophil subsets in PD-1 mMAB resistant patients was related to type I interferon, which may also be one of the influencing factors of reduced PD-1 mMAB sensitivity in esophageal cancer patients.

At present, the detection of PD-L1 is still mainly by immunohistochemistry, but there is a certain difficulty in tissue detection in specimen collection. Tumor cells can achieve immunotherapy resistance through a variety of mechanisms, such as T cell depletion leading to PD-1 blockade treatment resistance, or induction of tumor cells expressing PD-L1 leading to adaptive immune resistance. ESCC has entered the era of immunity. Biomarkers still need to be explored to screen the beneficiaries of immunotherapy. A large number of studies have reported that peripheral blood immune cells participate in the body’s anti-tumor immune response.

In this study, the effect of peripheral immune cell infiltration on the sensitivity of monoclonal antibody PD-1 in patients with esophageal cancer was thoroughly investigated by single cell sequencing technology, which provides a new idea for immunotherapy and effective biomarkers for esophageal cancer.

## Conclusion

In summary, we investigated the effect of peripheral blood immune cell infiltration on the sensitivity of PD-1 mMAB in ESCC by single cell sequencing. Through comprehensive analysis of the transcriptome characteristics of immune cells, the dynamic change of cell percentage, heterogeneity of cell subtypes and interactions between cells were explained to provide new understanding and potential therapeutic targets for the biological basis of ESCC immunotherapy.

## Data availability statement

The original contributions presented in the study are included in the article/**Supplementary Material**. Further inquiries can be directed to the corresponding author.

## Ethics statement

The studies involving human participants were reviewed and approved by Tianjin Medical University Cancer Institute and the hospital ethics committee. The patients/participants provided their written informed consent to participate in this study.

## Author contributions

TD, HW, CY, and MZ performed most of the experiments, analyzed data, and wrote the manuscript. ZJ, MB, TN, RL, JW, SG, LZ, and YB reviewed and edited the manuscript. HZ designed the experiments and edited the manuscript. HZ is the guarantor of this work and, as had full access to all of the data in the study and takes responsibility for the integrity of the data and the accuracy of the data analysis. All authors contributed to the article and approved the submitted version.

## Funding

This work was supported by grants from the National Natural Science Foundation of China (Nos. 82072664, 81974374, 82173125). The funders had no role in the study design, the data collection and analysis, the interpretation of the data, the writing of the report, and the decision to submit this article for publication.

## Acknowledgments

The tumor tissue samples of ESCC patients were obtained from Tianjin Medical University Cancer Institute and Hospital. We sincerely thank the Key Laboratory of Cancer, Cancer Prevention and Treatment, National Clinical Research Center of Tianjin Cancer Hospital, and the Clinical Cancer Research Center of Tianjin for providing us with experimental equipment, important reagents and valuable experimental suggestions.

## Conflict of interest

The authors declare that the research was conducted in the absence of any commercial or financial relationships that could be construed as a potential conflict of interest.

## Publisher’s note

All claims expressed in this article are solely those of the authors and do not necessarily represent those of their affiliated organizations, or those of the publisher, the editors and the reviewers. Any product that may be evaluated in this article, or claim that may be made by its manufacturer, is not guaranteed or endorsed by the publisher.
