# Supplementary material for: Single cell sequencing revealed the mechanism of PD-1 resistance affected by the expression profile of peripheral blood immune cells in ESCC
Source: Front Immunol. 2022 Nov 17;13:1004345. doi: 10.3389/fimmu.2022.1004345 (PMC9712746; doi:10.3389/fimmu.2022.1004345)

Supplementary Data

S1.

(A) Quasi - time analysis by cell type coloring diagram. Different colors indicate different cell types. This diagram can reflect the differentiation relationship of different cell types. (B) The abscissa is the pseudo time, and the ordinate is the density of cell number at different time points. Different colors indicate the density distribution of different cell types with pseudo-time. (C) A distribution diagram of each cell type alone in a quasi-temporal locus. (D) A distribution diagram of each sample in a quasi-temporal trajectory, where different colors indicate the cell types in each sample. (E) Display the dynamic changes of gene expression with the change of pseudo time. The abscissa from left to right is the time from small to large; the ordinate is the gene; each point represents the expression amount (mean value) of the gene at the specified pseudo time. (F) The heat map shows the differential genes that change with pseudo-time. (G) The first 8 genes (here the genes are ordered in reverse order according to q value) that change with pseudo-time (genes that are differentially expressed with pseudo-time). (H) Venn diagram of intersection of genes with reduced expression in PD-1 mMAB resistant ESCC patients. (I) Intersection Venn diagram of elevated gene expression in PD-1 mMAB ESCC patients. (J) TCGA database was used to analyze the prognosis and survival of patients with high and low expression of MT1X in ESCC patients. (K) Percentage of $CD8^+$ T cells in the sensitive and resistant group. (L) The proportion of MT2A, MT1E, MT1X positive $CD8^+$ T cells in $CD8^+$ T cells. *$p < 0.05$, **$p< 0.01$, respectively

S2

(A-C) KEGG enrichment analysis of monocyte Cluster 1-3. (D) GO enrichment analysis of monocyte Cluster 4. (E) KEGG enrichment analysis of monocyte Cluster 4.

S3

(A-C) KEGG enrichment analysis of neutrophil Cluster 1-4. (D) GO enrichment analysis of monocyte Cluster 5. (E) KEGG enrichment analysis of monocyte Cluster 5.

S4.

(A) The AUC matrix of cell types was calculated by means, and top 10 Regulon's CLUSTERING heat map of AUC matrix in each cell type. (B) A heat map of AUC matrix clustering in each cell is presented. (C-E) The regulator specific scatter plot of $CD8^+$ Te cell types in the ESCC_S, ESCC_D1, ESCC_D2 group was shown, highlighting the highest of the top 10 regulators. (F) Venn diagram shows the expression of each group of transcription factors.

Supplementary Figs. 1

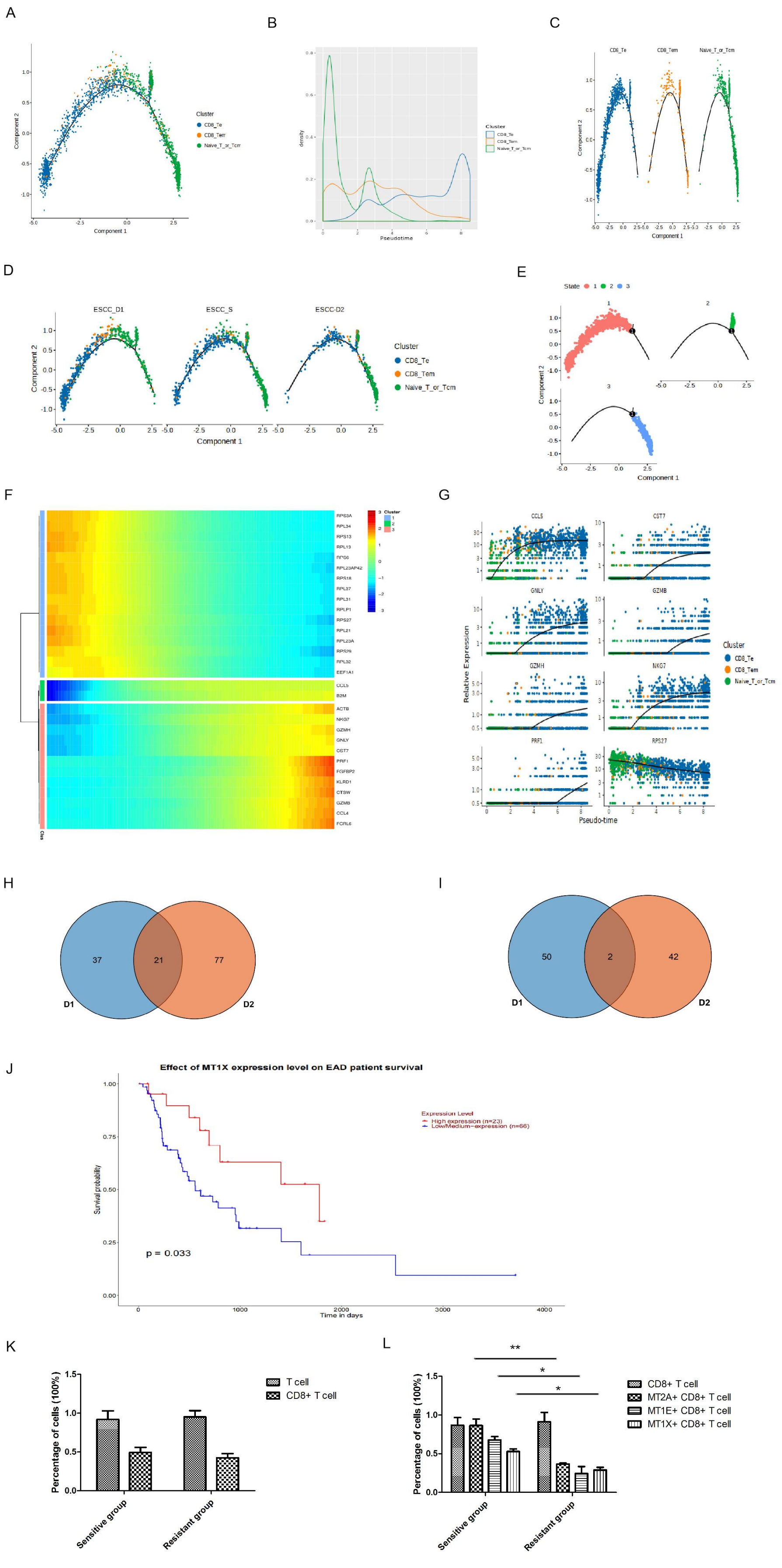

Supplementary Figs. 2

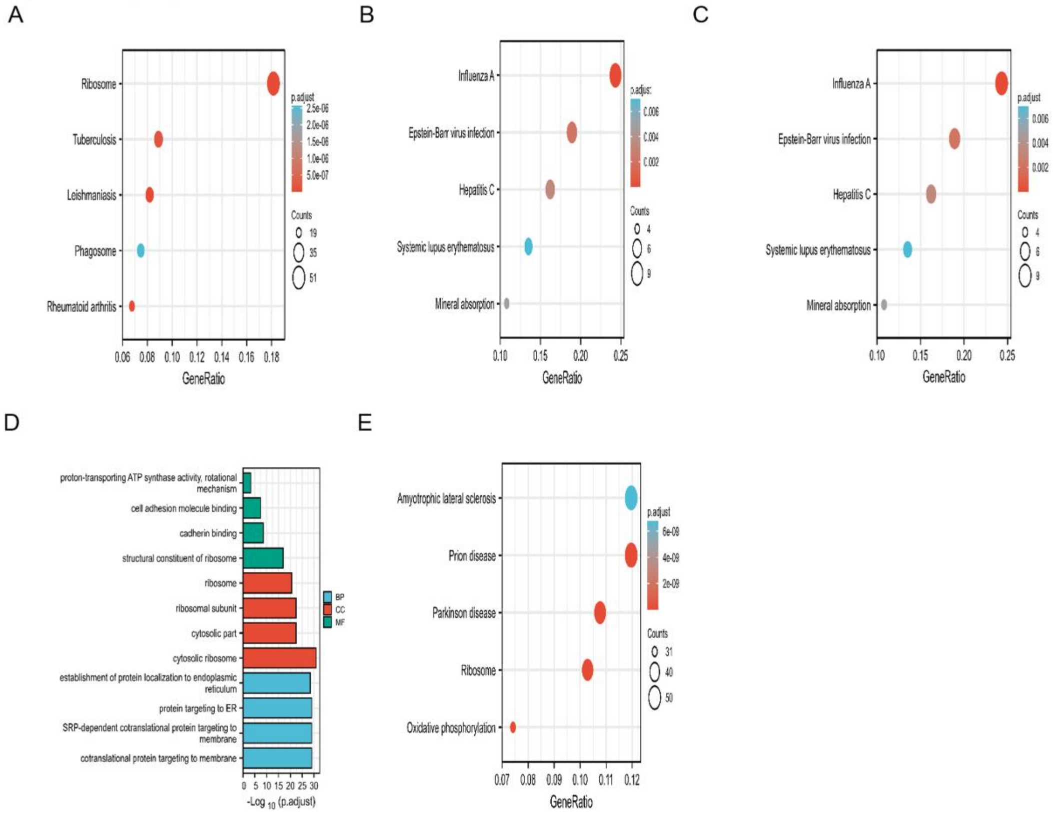

Supplementary Figs. 3

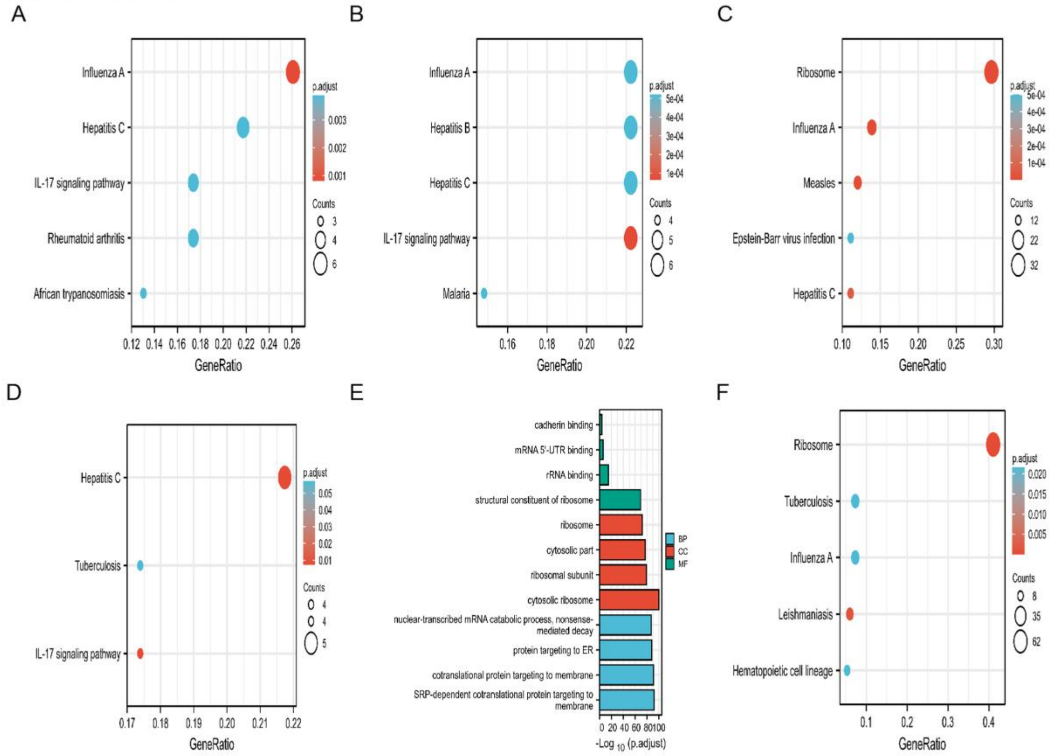

Supplementary Figs. 4

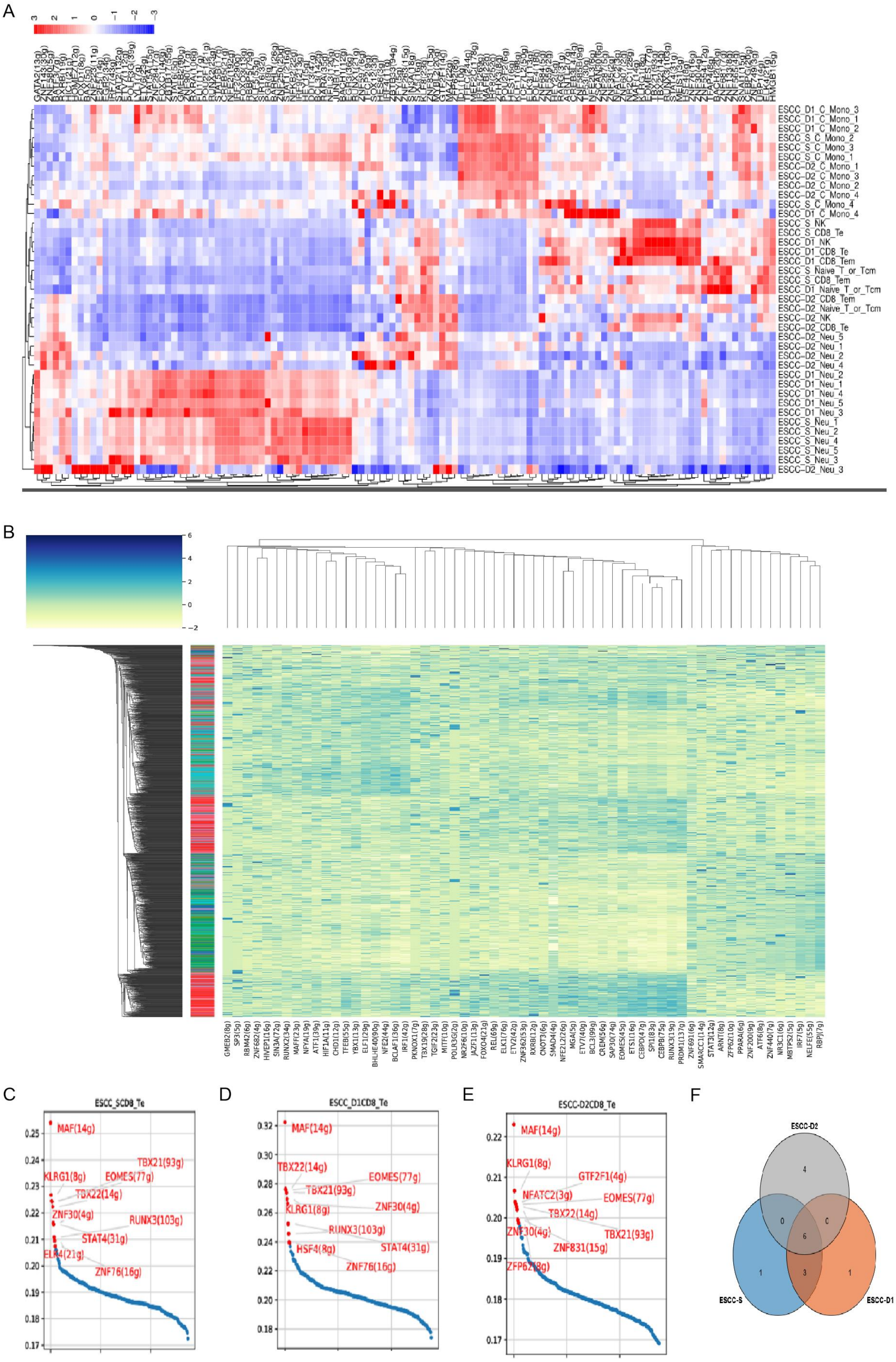

Supplement: Supplementary file 1 [file DataSheet_1.pdf]
